# Microbiome and its impact on fetal and neonatal brain development: current opinion in pediatrics

**DOI:** 10.1097/MCO.0000000000001028

**Published:** 2024-03-12

**Authors:** Nina M. Frerichs, Tim G.J. de Meij, Hendrik J. Niemarkt

**Affiliations:** a Amsterdam UMC, University of Amsterdam, Amsterdam Gastroenterology Endocrinology Metabolism Research Institute, Department of Pediatric Gastroenterology, Emma Children's Hospital Amsterdam, The Netherlands; b Amsterdam UMC, University of Amsterdam, Amsterdam Reproduction and Development Research Institute, Amsterdam The Netherlands; c Neonatal Intensive Care Unit, Máxima Medical Centre, Veldhoven; d Eindhoven University of Technology, Faculty of Electrical Engineering, Eindhoven, The Netherlands

**Keywords:** fetus, gut microbiota, gut-microbiota-brain axis, neonate, neurodevelopment

## Abstract

**Purpose of review:**

Emerging evidence suggests that the gut microbiota and its metabolites regulate neurodevelopment and cognitive functioning via a bi-directional communication system known as the microbiota-gut-brain axis (MGBA).

**Recent findings:**

The MGBA influences brain development and function via the hypothalamic-pituitary axis, the vagal nerve, immune signaling, bacterial production of neurotransmitters, and microbial metabolites like short-chain fatty acids, tryptophan derivatives, and bile acids. Animal studies show fetal neurodevelopment is mediated by maternal microbiota derivatives, immune activation, and diet. Furthermore, manipulation of the microbiota during critical windows of development, like antibiotic exposure and fecal microbiota transplantation, can affect cognitive functioning and behavior in mice. Evidence from human studies, particularly in preterm infants, also suggests that a disrupted gut microbiota colonization may negatively affect neurodevelopment. Early microbial signatures were linked to favorable and adverse neurodevelopmental outcomes.

**Summary:**

The link between the gut microbiota and the brain is evident. Future studies, including experimental studies, larger participant cohort studies with longitudinal analyses of microbes, their metabolites, and neurotransmitters, and randomized controlled trials are warranted to further elucidate the mechanisms of the MGBA. Identification of early, predictive microbial markers could pave the way for the development of novel early microbiota-based intervention strategies, such as targeted probiotics, and vaginal or fecal microbiota transplantation, aimed at improving infant neurodevelopment.

## INTRODUCTION

Advancements in sequencing techniques and metabolomics combined with bioinformatic pipelines refined our knowledge of microbiota composition and function [1]. As a result, the gut microbiota has been increasingly recognized for their role in influencing various aspects of health, including metabolism and immune function. During infancy, the gut microbiota has yet to be established and this dynamic maturation is influenced by many different factors like maternal microbiota, delivery mode, feeding pattern, gestational age, and medication [2,3]. Disturbance of the initial microbial colonization has been associated with a variety of diseases in the neonatal period, like necrotizing enterocolitis and sepsis, and beyond, like atopy, allergies, obesity, and auto-immune disorders [4–6].

There has been accumulating evidence that the gut microbiota and its metabolites also regulate various aspects of neurodevelopment and cognitive functioning throughout life. It is thought that the gut influences the brain and vice versa via an interplay of bi-directional pathways, mediated by gut microbiota: the microbiota-gut-brain axis (MGBA) [1]. The MGBA is considered to play a key role in infancy when significant stages of neurodevelopment take place [3,7], especially in preterm infants, as these are at an increased risk of both dysbiosis and impaired neurodevelopment [2]. Defining the critical windows of early-life microbiota colonization and its effect on neurodevelopment is an important emerging translational research question since this can form the basis for future microbiota-based interventions to preserve neurocognitive development and function in infants.

In this review, we discuss the mechanisms of the MGBA and provide an update on the emerging evidence regarding the connection between the maternal, fetal, and neonatal microbiota and neurocognitive development and functioning. Furthermore, we will discuss the therapeutic options to modulate the gut microbiota for preservation of neurodevelopment. 

**Box 1:**
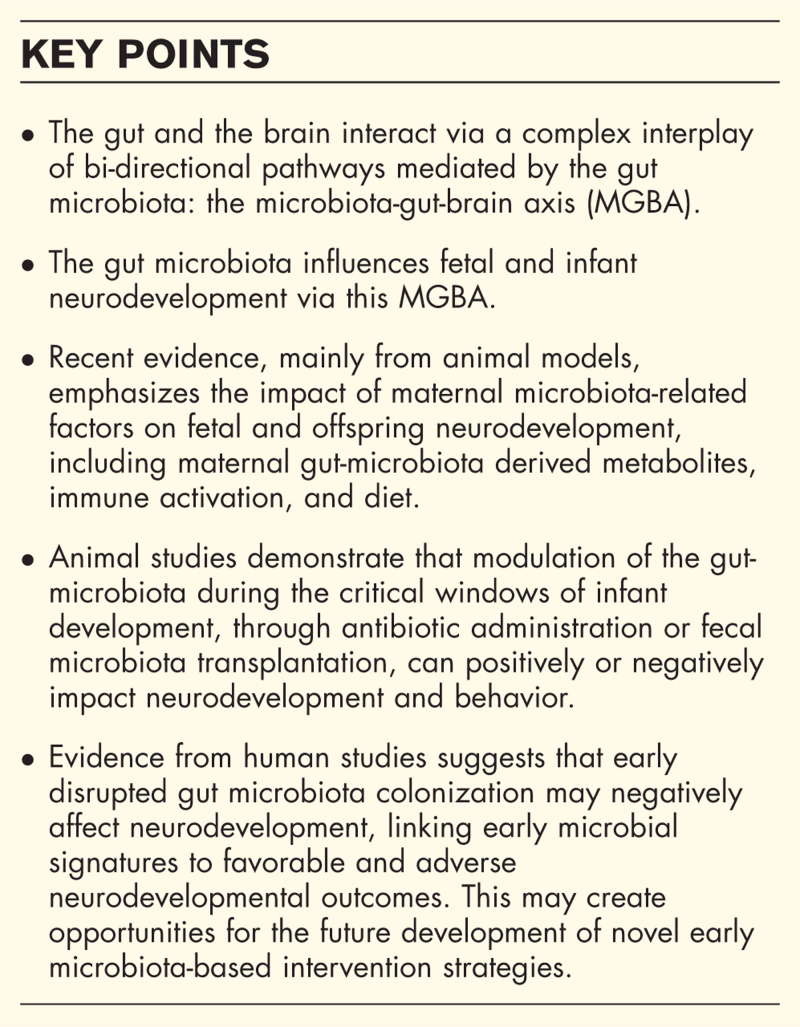
no caption available

## MICROBIOTA-GUT-BRAIN AXIS MECHANISMS

**FIGURE 1:**
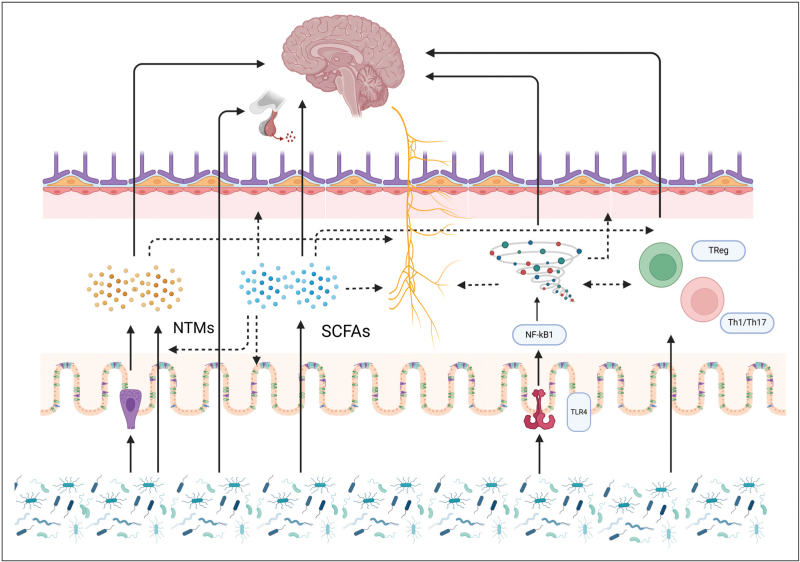
**Proposed mechanisms of the microbiota-gut-brain axis.** The microbiota-gut-brain axis is established by different pathways that communicate bi-directionally via different pathways in a complex interplay. This is a simplified depiction showing the most important pathways. Created with Biorender. NTMs, neurotransmitters; SCFAs, short-chain fatty acids; Th, T-helper cells; TLR4, toll-like receptor 4; Treg, regulatory T cells.

The vagal nerve is recognized as pivotal in facilitating bidirectional communication within the MGBA, constituting the primary conduit for microbiota-derived signals to interface with the brain. Although the vagal nerve itself is not in direct contact with the gut microbiota [8], it serves as a receptor for signals transmitted via microbial metabolites, inflammatory processes, or neuroendocrine cells which are mediated by gut microbiota [2,9] (Fig. 1). The microbiota exerts regulatory control over the neuroendocrine hypothalamic-pituitary axis (HPA). Experimentally induced gut dysbiosis modulates HPA-mediated cortisol release and consequent behavioral outcomes [1,9].

The gut microbiota produces a plethora of metabolites such as short-chain fatty acids (SCFAs), tryptophan metabolites, and biliary acids. SCFAs, such as butyrate, propionate, and acetate, result from anaerobic microbial fermentation of indigestible polysaccharides in the colon [10]. SCFAs contribute to the maintenance and modulation of both gut and blood—brain barrier (BBB) integrity [11,12^▪▪^]. They can directly affect the brain compromising the BBB, binding to local receptors, inducing epigenetic modifications, contributing to brain homeostasis, and modulating neuro inflammation [13]. For instance, butyrate can possess direct anti-inflammatory effects on oligodendrocytes which are crucial for early brain development [11,14]. SCFAs can enhance regulatory T cell (Treg) function, vital for immune tolerance, and activate the sympathetic nervous system and vagal nerve [11,15]. Indole, a tryptophan derivative, exhibits beneficial effects on neuro inflammation, nerve signal transduction, and gut and BBB integrity [9,16]. Lastly, biliary acids play a role in the brain and HPA axis through specific local receptors [9].

Various neurotransmitters, and their compounds, such as serotonin (derived from tryptophan), dopamine, norepinephrine, histamine, and gamma-aminobutyric acid (GABA), can be directly produced by gut microbiota or indirectly through the stimulation of entero-endocrine cells in the gut epithelium. These processes can occur under the stimulation of SCFAs. For example, *Lactobacilli*, *Bifidobacteriae*, and *Bacteroidetes* are capable of producing GABA [8,15], while *Escherichia coli* (*E. coli*) and specific strains of *Lactobacillus* can contribute to serotonin production. These neurotransmitters can exert local effects by stimulating the vagal nerve or gut peristalsis [8], but can also traverse the SCFA weakened gut and BBB, exerting a more direct impact on the brain [11].

The gut microbiota can also influence the brain through immune signaling. Under eubiosis, immune cells in the gut-associated lymphoid tissue differentiate into anti-inflammatory Treg cells producing cytokines like interleukin (IL)-10 and TGF-β [17]. During dysbiosis, lymphocytes differentiate towards the Th1/Th17-subtype, resulting in the production of pro-inflammatory cytokines such as IL-2, IL-12, and TNF-α [17]. Furthermore, lipopolysaccharide (LPS) containing bacteria can induce an inflammatory cascade by TLR 4 signaling, with NF-kB induced release of pro-inflammatory cytokines such as TNF-α, IL-1β, and IL-6. These can directly affect the brain, by the weakening of the BBB, or via pathogenic signaling through the vagal nerve [11]. The resulting inflammatory processes damage the oligodendrocytes, pivotal for early brain development.

## PRENATAL GUT MICROBIOTA INFLUENCES FETAL NEURODEVELOPMENT

Neonates are not immunologically naive but primed to respond to exposure to environmental and microbial stimuli. Consequently, there has been debate about the presence of a fetal gut microbiome [18]. However, human and murine studies failed to provide evidence for the existence of a fetal gut microbiota [18,19]. More recently, it was postulated that the maternal gut microbiota influences fetal immune and neurodevelopment indirectly through microbiota-derived metabolites [20]. These metabolites cross the placenta during pregnancy, provoking a fetal immune response [12^▪▪^]. This is supported by studies demonstrating the human fetal intestine to exhibit a diverse metabolome at only 12 weeks gestation, with enrichment of metabolites and metabolic pathways associated with neurodevelopment [21]. Additionally, comparative studies between germ-free mice and specific-pathogen free (SPF) mice revealed higher levels of microbial-originating metabolites in SPF mice, showing the lack of a maternal microbiota can affect metabolite levels in fetal organs [22]. Furthermore, depletion of the maternal microbiome in germ-free mice impairs fetal thalamocortical axonogenesis, while maternal colonization with *Clostridia*-dominant spore-forming bacteria increases levels of specific bacterial metabolites, thereby preventing these axonal defects [23]. Despite these findings, the mechanisms underlying transportation across the placenta remain unclear, necessitating further exploration.

Maternal immune activation (MIA) during pregnancy can have detrimental impacts on neurodevelopment [1,20]. MIA presumably induces cytokine production through the activation of Th17 cells via the microbiota. During induced MIA in mice, the level of maternal IL-17a produced by Th17 cells increased the risk of offspring developing autism spectrum disorder (ASD), which was mediated by the microbiota [24].

Maternal obesity before pregnancy and a high-fat diet (HFD) during pregnancy and lactation have been associated with multigenerational neurodevelopmental delay and social dysfunction [25,26]. In the first offspring, maternal supplementation of milk fat globule membrane (MFGM) during lactation promoted neurogenesis in the hippocampus and neurobehavioral development by modulation of gut microbiota. This modulation resulted in the downregulation of pro-inflammatory bacteria such as *E. shigella* and *Enterococcus*, and upregulation of bacteria with anti-inflammatory and antiobesity properties, such as *Akkermansia* and *Lactobacillus*. Consequently, neuroinflammation was alleviated by reduced levels of microbiota-correlated pro-inflammatory factors, such as LPS and IL-1β [25]. In the second offspring, social dysfunction was restored through supplementation with *Limosilactobacillus reuteri*, which facilitated the expansion of SCFA-producing bacteria in their gut microbiota [26].

## IMPACT OF GUT MICROBIOTA DURING CRITICAL WINDOWS OF DEVELOPMENT

### Recent evidence in animals

Animal studies demonstrate that induced microbiota alterations during the critical developmental window affect neurodevelopment and behavior. Lynch *et al.*[27] explored the effects of early-life *antibiotic administration* during critical developmental windows in mice, revealing a dramatic disruption of the cecal microbiota of adolescent mice. This disruption incorporated increases of *Escherichia/Shigella*, *Staphylococcus*, and *Clostridioides,* while key producers of SCFAs as *Alistipes, Bacteroides, Odoribacter*, and *Lachnospiracea NK4A136,* were decreased. Furthermore, alterations of microglia morphology were observed in the basolateral amygdala of adolescent mice, suggesting that short-term targeted disruption of the developing gut microbiota can have enduring effects on microglia maturation [27].

Fecal microbiota transplantation of human feces into germ-free mice elegantly shows the transferability of cognitive phenotypes through the gut microbiota. Recently, pregnant germ-free mice were colonized with fecal microbiota obtained from preterm infants of various postmenstrual ages (PMA), ranging from 27 to 34 weeks. Colonization with fecal microbiota of infants with higher PMA improved associative fear learning and memory in adult mice. Thirteen upregulated fecal and serum metabolites, linked to brain function, correlated with microbial maturation-associated cognitive improvements [28]. Another study involved transplanting feces from 6-month-old infants, differing in cognition scores, into germ-free mice. Mice transplanted with feces of infants with above-median cognition scores exhibited better memory functions. The gut microbiota of these mice was enriched in species belonging to the genera *Phocaeicola, Bacteroides,* and *Bifidobacterium*[29]. An FMT study from 5-year-old children with varying cognitive scoring into germ-free mice confirmed cognitive phenotype transferability but also identified three unique fecal metabolites – xanthine, formate, and mannose – with long-lasting strong positive associations with high cognitive performance phenotypes [30].

Several studies explored the effects of *diet-induced microbial alterations* on brain development and behavior. In rats prenatally exposed to caffeine for induced intrauterine growth restriction, a HFD led to ASD like behavior [31]. *E. coli* was significantly increased in the gut microbiota of these rats, with increased levels of IL-17A in the colon, serum, and hippocampal regions. The *E. coli* and IL-17A concentrations in the hippocampal regions were closely correlated with ASD symptoms. *E. coli* strain transplantation in control rats led to spontaneous development of ASD-like manifestations and similar increases in IL-17A [31]. Contrary to HFD, supplementation of nucleotides, found in human milk, enhanced neuro-maturation in the prefrontal cortex and hippocampus in rats. This was mediated by gut microbiota composition and function and correlated to neurodevelopmental phenotypes [32].

### Recent evidence in humans

Comparable to murine studies, the maternal gut microbiota influences neonatal neurodevelopment in humans. In a recent study by Sun *et al*. [33^▪^], maternal microbiota seemed more relevant to the offspring's neurodevelopment than the infants gut microbiota, with *Fusobacteria* as a key player, increasing later motor skills when found in maternal gut microbiota and decreasing motor skills when found in infants.

Recently, the correlations between early human gut colonization and neurodevelopmental outcomes. In 44 term infants, associations were observed between the gut microbiota and early cognitive development. A successful Point-and-Gaze attention test was associated with increased Actinobacteria and reduced Firmicutes on the phylum level, and increased *Bifidobacterium* and *Eggerthella* and reduced *Hungatella* and *Streptococcus* on the genus level [34]. Another recent study using Random Forest modeling demonstrated that gut microbial species in the first year of life predict future cognitive functioning. *Klebsiella* spp., *E. coli*, and *Bifidobacterium* spp. were important predictors of cognitive functioning. In addition, RF modeling on bacterial species predicted the size of brain regions on MRI [35]. A recent randomized controlled trial (RCT) investigated the impact of vaginal microbiota transfer (VMT) on neurodevelopmental outcomes in 68 infants born via cesarean-section. VMT infants showed significantly higher neurodevelopmental scores (as measured by Ages-and-Stages-Questionnaire-3 (ASQ-3) compared to placebo at 6 months of age. Furthermore, VMT was associated with faster gut microbiota maturation and with modulation of specific fecal metabolites and metabolic functions within the first 6 weeks of life [36^▪▪^].

More evidence is available on preterm infants. In 24 very low birth weight (VLBW) infants, neurodevelopment was examined by the Battelle Development Inventory-2 Screening Test (BDI-2ST) at 2 and 4 years of age [37]. Correlations between early microbial diversity and specific microbial amplicon sequence variants and BDI-2ST subscales such as cognition, adaptive behavior, and communication were observed, even after adjusting for gestational age, birth weight, and antibiotic exposure. *Bifidobacterium* presence appeared to be a pivotal element in infants not requiring neurodevelopmental referral at 2 years. In accordance, VLBW infants (*n* = 27) with adverse neurological outcomes at 24 months corrected age (MCA), measured with the revised Griffiths Mental Development Scale, were characterized by early deficiency of *Bifidobacterium*[38]. Seki *et al*. [39] showed early overgrowth of *Klebsiella* ssp. was associated with a pro-inflammatory T cell response and seemed highly predictive for brain damage as identified by MRI at term age in 60 extremely preterm infants. A disturbed gut-microbiota-immune-brain axis may induce or worsen brain injury in extremely preterm infants [39]. Zhang *et al*. [39] evaluated the relationship between gut microbiota composition in seventy-seven preterm infants at four weeks of age and neurodevelopmental outcomes up to 6 MCA (ASQ-3). Beta diversity was linked with gross motor scores at 1, 3, and 6 MCA, communication scores at 3 MCA, and fine motor scores at 6 MCA [40^▪^]. Comparably to earlier findings [35,39], the relative abundance of *Klebsiella* ssp. was negatively associated with gross motor scores from 1 to 6 MCA, while the relative abundance of *Lactobacillus* exhibited a positive association [35,39,40^▪^].

## MODULATING GUT MICROBIOTA FOR NEURODEVELOPMENT

Identification of early, predictive microbial markers holds the potential for the development of novel intervention strategies to enhance neurodevelopment. Proposed microbiota-based interventions include human milk, probiotics, VMT, FMT, and avoidance of certain medications.

Human milk, rich in human milk oligosaccharides (HMO) and MFGM, promotes the growth of beneficial bacteria such as *Bifidobacterium* spp. and *Bacteroides*[14]. In term infants, HMO exposure is positively associated with cognitive, language, and motor skill domains between 18 and 24 months of age, however, this effect was not seen in preterm infants [41]. A recent RCT showed improved cognitive function at 5.5 years of age in MFGM-supplemented term infants receiving formula milk [42]. However, another RCT comparing human donor milk to formula feeding in preterm infants unable to receive sufficient mothers’ milk did not show differences in neurocognitive outcome at 2 years of age [43]. Although mothers’ own milk is presumed to have a beneficial impact on neurodevelopment, this has to date only been proven in retrospective studies, as RCT are deemed unethical [44]. Notably, the beneficial effects of mothers’ milk on neurodevelopment are various and go beyond gut microbiota.

Judicious antibiotic use in preterm infants, given their frequent empirical antibiotic treatment, can positively impact gut microbiota and subsequent neurocognitive outcomes [45]. In an observational study – with inherent limitations and therefore not proving causation – we observed that prolonged empirical antibiotic treatment in preterm infants was associated with below-average gross-motor development at 24 MCA [46]. The avoidance of acid suppressants, known to alter gut microbiota, could therefore be beneficial for preserving neurocognitive outcomes, as a recent study showed the use of acid suppressants to be associated with adverse neurocognitive outcomes [47]. However, it was not confirmed that this effect was mediated by changes in gut microbiota.

Probiotic administration in (preterm) infants has shown mixed results on neurocognition; two RCTs suggest long-term benefits in neurocognitive functioning in term infants, but larger RCTs are needed for robust evidence [48,49]. Recently, maternal VMT has shown promise to restore microbiota maturation and lead to better neurocognitive outcomes at 6 months of age in term infants born with cesarean-section [36^▪▪^], while safety and long-term results of maternal FMT to (preterm) infants are being explored in the MT-SECFLORE and PREFLORE trials [50,51].

## CONCLUSION

Increasing evidence in animals and humans suggests that disrupted gut microbiota colonization can negatively affect MGBA development, thereby affecting brain development, neurocognition, and behavior. In humans, early microbial signatures have been associated with adverse neurodevelopmental outcomes (*Klebsiella* spp.) while an increased abundance of *Bifidobacterium* show beneficial effects. Future studies, including larger cohort studies with longitudinal analyses of microbes, their metabolites, and neurotransmitters, can further elucidate the mechanisms of the MGBA. Well-designed experimental studies and RCTs are necessary to prove whether the associations between the MGBA and brain development, neurocognition, and behavior are causative or attributable to other factors. Implementation of a combination strategy of the discussed interventions holds the potential for improving neonatal neurodevelopmental outcomes through MGBA modulation.

## Acknowledgements

*None.*

### Financial support and sponsorship

*None to declare.*

### Conflicts of interest

*N.F., T.dM., and H.N. have received an unrestricted grant from Nutricia Benelux Corporation unrelated to this submitted work.*

## REFERENCES AND RECOMMENDED READING

Papers of particular interest, published within the annual period of review, have been highlighted as:

▪ of special interest
▪▪ of outstanding interest
